# The rise in climate change-induced federal fishery disasters in the United States

**DOI:** 10.7717/peerj.11186

**Published:** 2021-04-22

**Authors:** Lyall Bellquist, Vienna Saccomanno, Brice X. Semmens, Mary Gleason, Jono Wilson

**Affiliations:** 1 California Oceans Program, The Nature Conservancy, San Diego, CA, United States of America; 2 Scripps Institution of Oceanography, University of California, San Diego, La Jolla, CA, United States of America; 3 California Oceans Program, The Nature Conservancy, Los Angeles, CA, United States of America; 4 California Oceans Program, The Nature Conservancy, Monterey, CA, United States of America; 5 California Oceans Program, The Nature Conservancy, Santa Barbara, CA, United States of America; 6 Bren School of Environmental Science & Management, University of California, Santa Barbara, Santa Barbara, CA, United States of America

**Keywords:** Extreme environmental events, Fishing communities, Marine heatwaves, Fisheries economics, Federal fishery disasters, Climate ready fisheries management

## Abstract

Commercial, recreational, and indigenous fisheries are critical to coastal economies and communities in the United States. For over three decades, the federal government has formally recognized the impact of fishery disasters via federal declarations. Despite these impacts, national syntheses of the dynamics, impacts, and causes of fishery disasters are lacking. We developed a nationwide Federal Fishery Disaster database using National Oceanic and Atmospheric Administration (NOAA) fishery disaster declarations and fishery revenue data. From 1989-2020, there were 71 federally approved fishery disasters (eleven are pending), which spanned every federal fisheries management region and coastal state in the country. To date, we estimate fishery disasters resulted in $2B (2019 USD) in Congressional allocations, and an additional, conservative estimate of $3.2B (2019 USD) in direct revenue loss. Despite this scale of impact, the disaster assistance process is largely ad hoc and lacks sufficient detail to properly assess allocation fairness and benefit. Nonetheless, fishery disasters increased in frequency over time, and the causes of disasters included a broad range of anthropogenic and environmental factors, with a recent shift to disasters now almost exclusively caused by extreme environmental events (e.g., marine heatwaves, hurricanes, and harmful algal blooms). Nationwide, 84.5% of fishery disasters were either partially or entirely attributed to extreme environmental events. As climate change drives higher rates of such extreme events, and as natural disaster assistance requests reach an all-time high, the federal system for fisheries disaster declaration and mitigation must evolve in order to effectively protect both fisheries sustainability and societal benefit.

## Introduction

Marine fisheries represent an important part of the United States (US) economy, employment, and food security, generating $244B in annual revenue, providing 1.7M part- and full-time jobs (NOAA, 2020a), and supporting a centuries-long history of community, cultural, and indigenous heritage. While overcapitalization, data limitations, and mismanagement have led to historical fishery declines, more recent fisheries science and management advancements have been achieved nationwide (Methot Jr et al., 2013; National Research Council, 2014; NOAA, 2016), including improved stock status of US federally managed fisheries and rebuilding of overfished species (NOAA, 2020b). However, climate change and extreme environmental events (e.g., marine heatwaves) are adding significant challenges to an already underfunded and overburdened fisheries management system (Hoegh-Guldberg & Bruno, 2010; Di Lorenzo & Mantua, 2016; Wernberg et al., 2016; Free et al., 2019; Belhabib et al., 2018), threatening both national food security and the balance between healthy economies and ecosystems (Ding et al., 2017). The COVID-19 pandemic has also added a new suite of impacts (Bennett et al., 2020), leading to the highest annual number of natural disasters declared in 2020 in the history of the Federal Emergency Management Agency (FEMA, 2020).

Recent improvements in our understanding of linkages between fisheries, ecosystems, climate, communities, and economies indicate that disaster impacts can have rapid and lasting effects (Hoegh-Guldberg & Bruno, 2010; Wernberg et al., 2016). When US fisheries are impacted beyond federally designated economic thresholds, the US Secretary of Commerce can formally declare a Federal Fishery Disaster (Upton, 2019), which results in financial compensation for impacted fishing communities. Fishery disaster determinations represent a federal response to anthropogenic and/or environmental impacts that spread throughout entire fisheries supply chains, from the resource itself, to fishing communities, families, and to associated fishing, seafood, clothing/equipment, marine, manufacturing, and hospitality industries (Upton, 2019; Oliver, 2019). Fishery disaster declarations are also largely reactive by design, meaning that when disaster assistance is requested (let alone disbursed), the disaster impacts have already been realized.

Fishery disasters are approved on a case-by-case basis by the US Secretary of Commerce under the jurisdiction of sec312(a) and 315 of the Magnuson-Stevens Fishery Conservation and Management Act (MSA; 16 U.S.C. §1861(a) and §1864,), as well as sec308(b and d) of the Interjurisdictional Fisheries Act (IFA; 16 U.S.C. §4107). Fishery disaster requests are typically filed by either state governor(s) or elected or duly appointed representative(s) of affected indigenous fishing communities. For a disaster request to be approved, three fundamental criteria must be met: (1) there must be an identified state, federal, or Native American fishery resource disaster associated with either species population decline or loss of fishing infrastructure, (2) it must have an “allowable cause” (Upton, 2019), and (3) there must be economic impact resulting from the disaster. If it is concluded that a fishery disaster occurred, then the economic data are analyzed to determine whether the disaster led to a commercial fishery failure. Commercial fishery failures are declared if revenue losses are greater than 80% relative to the mean annual revenue over the most recent five-year period (Upton, 2019; Oliver, 2019). Revenue losses of less than 35% are generally not eligible for assistance, and losses between 35–80% receive further evaluation. An additional federal designation, a catastrophic regional fishery disaster, may also be declared if the following criteria are met: (1) if either a commercial fishery failure or fishery resource disaster are declared by the US Secretary of Commerce, (2) if the event affects more than one state or fishery managed by a regional council or Interstate Fishery Commission (IFC), and (3) if the event results in economic losses to coastal or fishing communities (Upton, 2019). If a commercial fishery failure is determined, the US Congress may appropriate assistance funds to the impacted states or communities. These groups must then create a spending plan that is reviewed by the National Oceanic and Atmospheric Administration (NOAA) before assistance is administered. Under the MSA, the federal government cannot allocate greater than 75% of the cost of assistance activities toward individual disaster assistance, with the remaining 25% typically covered by state or regional entities. The complexity of the process and administrative steps required often result in disbursement of funds occurring years after the disaster occurred (Upton, 2019; Oliver, 2019).

**Figure 1:**
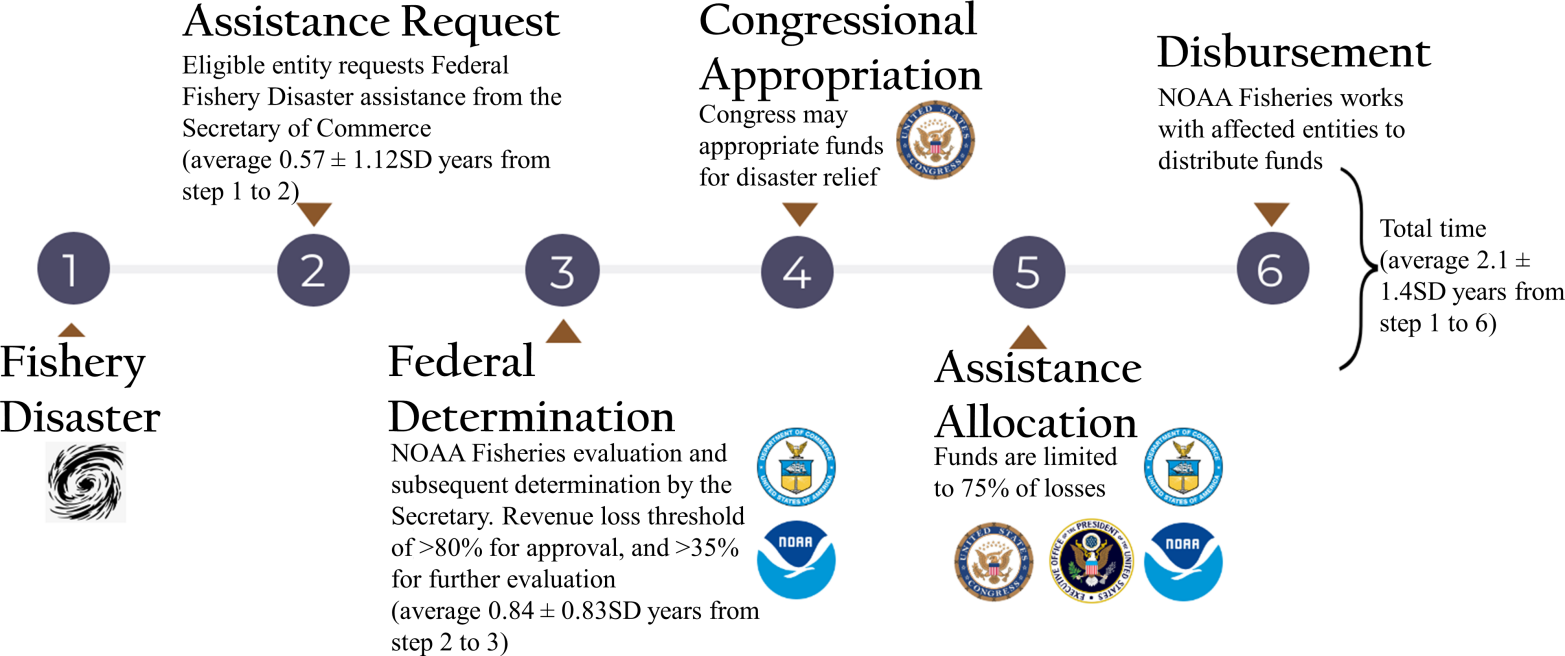
Federal fishery disaster request, determination, and administration process. Agency seals represent institutional involvement during each step of the disaster assistance process. Time periods in parentheses represent the mean (±SD) duration required for each step to occur. While the total implementation time is known (shown after step 6), some individual time steps do not have associated duration information.

Federal Fishery Disaster review and assistance implementation timelines follow a six-step process: (1) disaster event, (2) request for assistance, (3) analysis and determination, (4) appropriation, (5) allocation, and (6) a grant out for assistance (Fig. 1). Disaster assistance can be structured in numerous ways, generally with fishing communities as the primary intended beneficiaries. Assistance can be appropriated by US Congress and routed through IFCs or individual states, and then allocated directly to fishers and fishing communities through grants, job training, cooperative agreements, contracts, or low-interest loans (Upton, 2019). Assistance can also be administered in a more proactive context to fisheries science or management programs, including data collection, compensated reduction in capacity, or resource restoration projects. This administrative process can be contentious since federal decision-making may be unclear, data driving disaster determinations are not publicly reported, impacted individuals or entities may be difficult to identify, and the degree of economic impacts may be difficult to quantify.

The thirty-year history and national scale of fishery disasters in the US indicate a clear need for a nationwide synthesis that can allow fisheries scientists, managers, and policymakers to advance a more adaptive fisheries management approach in a new disaster-specific context. To date, federal fisheries science and management have focused largely on reducing the number of stocks that are overfished or experiencing overfishing. Meanwhile, US Federal Fishery Disasters have been processed on a case-by-case basis with a lack of peer-reviewed science dedicated to comprehensive learning across all disasters. Our aim was to produce a national synthesis that answers how the frequency and causes of disasters have changed since the inception of the assistance program, and to estimate the economic impacts of disasters at both national and regional scales. This will not only inform disaster-related fishery management and policy decisions, but it will expand on our understanding that overfished/overfishing stock metrics, such as the NOAA Fish Stock Sustainability Index (FSSI), should be the primary metric with which we evaluate national fishery management success. To illustrate the scale and trends of disasters and their associated economic impacts, we developed a national and regional database of all Federal Fishery Disasters in the US and synthesized the associated spatial and temporal data reported in US federal disaster records. We used the frequency of disasters and common details across federal disaster requests and approvals to analyze temporal trends and to create a standardized metric for annual and region-specific disaster impacts. We estimated economic impacts based on Congressional allocations for disaster assistance as well as direct fishing revenue loss using NOAA and state-level landings and revenue data. In so doing, we highlight a recent shift in disaster frequency and causes; we discuss inefficiencies in the request, determination, and implementation processes; and we open a discussion for more proactive solutions to managing fishery disasters. This is especially critical when federal disaster assistance requests reached an all-time high (FEMA, 2020), and when climate change impacts are emerging as a significant threat to coastal fisheries, communities, economies, and ecosystems (Hoegh-Guldberg & Bruno, 2010; Pecl et al., 2017; Lotze et al., 2019).

## Materials & Methods

**Figure 2:**
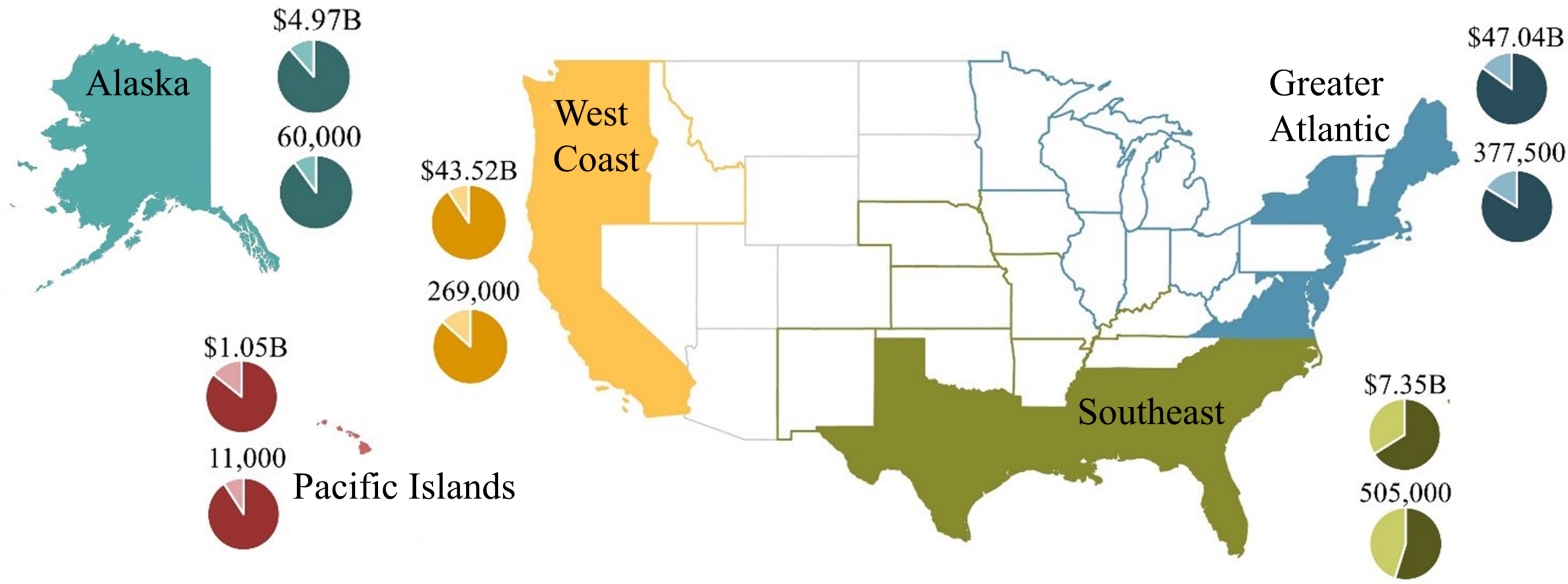
Five US federal fisheries management regions (Alaska, West Coast, Pacific Islands, Greater Atlantic, and Southeast) represented in the fishery disaster database. In each region, pie charts represent annual commercial and recreational fisheries sales impacts (top pie chart) and total number of jobs (bottom pie chart) to illustrate regional contributions to the US economy. The dark shaded region in each pie chart represents commercial fishery values, and the light shaded region represents recreational fishery values (source: NOAA, 2020a).

We developed a US Federal Fishery Disaster database separated by federal management regions (Fig. 2) using the NOAA Fishery Disaster Assistance online portal produced by the Office of Sustainable Fisheries (NOAA, 2020c). In this portal, fishery disaster determinations were listed with specific disaster information, including (when available) the assistance request letter, federal decision letter, press release, and funding authority. Most disaster records included the affected state(s), year(s), fishery(ies), federal management region, specific area, individual requestor(s), request date and letter, determination status, press release, determination authority and letter, funding authority, cause of the disaster, and appropriation amount. These records, combined with state and federal landings and revenue data, were used to examine trends in disaster frequency and impacts, causes of disasters, federal funding allocation, and direct revenue impacts. We carried out all statistical analyses in the statistical software environment R. All Federal Fishery Disaster data used in these analyses are available at https://www.fisheries.noaa.gov/national/funding-and-financial-services/fishery-disaster-determinations, and all fishery landings and revenue data are available via the NOAA Fisheries One Stop Shop (FOSS) website (NOAA, 2020d). All code for the analyses are available in the following GitHub repository: https://github.com/vrsaccomanno/federal-fish-disasters.git.

We assessed evidence for change in disaster frequency across the 30-year time series in two ways: (1) we assessed trends in the frequency of disaster determinations per year (each of which may specify multi-year disasters; e.g., a disaster declared in 2002 may specify disaster impact years 1999-2002), and (2) we assessed trends in the number of disasters per year. In both cases, we fit the following Poisson regression generalized linear model (GLM) with a log link, and scaled (Z-scored) year as a fixed continuous effect to model the count of disaster determinations or ongoing disasters per year. Given that the mean and variance for both response variables are approximately equivalent, we feel the assumption of Poisson distributed data is appropriate (we note, however, that findings from a negative binomial instance of the same models were essentially identical). The frequency of disasters will likely vary by management zone (Alaska, Greater Atlantic, Pacific Islands, Southeast, West Coast) in part because different regions are more likely to experience different types of disasters (e.g., hurricanes are not likely to impact the West Coast and Alaska regions). To address the potential for non-independence between zones, we also fit the following Poisson generalized linear mixed models (GLMM) with a fixed continuous effect of year (again, scaled) and random categorical effects of management zones, using the *glmer* function in the R library lme4 (Bates et al., 2015). Model formulations for both the GLM and GLMM models, including parameter estimates, are provided in Table 1.

**Table 1:** Mixed and fixed effects formulations and parameter estimates for models of disasteroccurrence and causes across years. Note that prior to model fitting, we transformed the vector of years into Z-scores in order to aid parameter estimation (subtracted all years by the mean of years, then divided by the standard deviation of years). The model formulation column reflects model specification in the R programming environment. In each of the parameter columns (Intercept and Year Effect) we report mean estimates with standard errors in parentheses. For the Year Effect parameter (parameter of interest) we report *p*-values. The RE Variance column reports the model estimated random effects variance, when appropriate. The final column reports Akaike information criterion (AIC) values for each model instance.

Response	Model method	Model formulation	Intercept(s)	Year effect	RE variance	AIC
Disaster Determinations	Poisson GLMM	∼Determination.Year + (1—Management.Zone)	−0.36 (0.14)	0.42 (0.12), *p* < 0.0008	0.014	226.4
Disaster Determinations	Poisson GLM	∼Determination.Year	0.94 (0.13)	0.38 (0.13), *p* < 0.003	NA	113.7
Ongoing Disasters	Poisson GLMM	∼Impact.Year + (1—Management.Zone)	−0.36 (0.14)	0.26 (0.10), *p* < 0.01	0.01	259.2
Ongoing Disasters	Poisson GLM	∼Impact.Year	0.94 (0.13)	0.38 (0.11), *p* < 0.0003	NA	121.3
Disaster Cause	Ordinal logistic GLMM	∼Determination.Year + (1—Management.Zone)	NAnthropogenic—Combination of Both: - 2.1 (0.54) Combination of Both—Environmental: - 0.28 (0.45)	0.94 (0.27), *p* < 0.0006	0.51	130.1
Disaster Cause	Ordinal logistic GLM	∼Determination.Year	Anthropogenic—Combination of Both: - 1.94 (0.36) Combination of Both—Environmental: - 0.281 (0.26)	0.87 (0.25), *p* < 0.0004	NA	130.5

Fishery disaster causes were assigned based on information provided in formal federal declarations, request letters, determination letters, press releases, federal fisheries reports, and/or primary literature sources. These causes, such as Harmful Algal Blooms (HABs), hurricanes, warming events, or oil spills, were then condensed into three ordered categories: environmental, combination of environmental and anthropogenic, and anthropogenic. Disasters resulting from hurricanes or HABs were labeled as environmental, but disasters resulting from overfishing or oil spills were labeled as anthropogenic. Because the publicly reported federal disaster declarations did not often include a cited rationale for cause determinations, with many causes labeled arbitrarily as ‘natural disaster of unknown causes,’ we used peer-reviewed fisheries literature and federal fisheries reports as additional sources for cause classifications. This allowed a more detailed understanding and documentation of the causes of each disaster. For example, causes of West Coast salmon fishery disasters were typically classified as a combination of both environmental (e.g., drought or warming) and anthropogenic (e.g., overfishing, habitat loss, fish passage barriers) factors due to the wide variety of well documented impacts on salmon stocks in each case. Once causes were assigned, disaster records were then separated by cause, year, and management region to highlight not only reasons why disasters are occurring, but also whether the reasons behind them are changing over time. To assess trends in the frequency of these categorical assignment through time, we performed an ordinal logistic regression with the category of each disaster as the response, and year as a fixed continuous effect. As above, we also fit a mixed effects ordinal logistic regression with fixed effect of year and random effects of management zone. We fit the fixed effects model using the *polr* function in the R library MASS (Ripley et al., 2013), and the mixed effect model using the *clmm2* function in the R library ordinal (Christensen, 2019).

Economic impact associated with each fishery disaster was measured using the federal assistance dollar amount allocated by Congress, as well as estimates of the direct fishing revenue impact. All dollar amounts were converted from nominal to real dollars (2019 USD) using an inflation multiplier from the US Bureau of Labor Statistics CPI inflation Calculator for the year in which the disaster occurred (U.S. Bureau of Labor Statistics, 2020). The federal assistance amount was sourced from the NOAA Fishery Disaster Assistance online portal, although federal disaster number 19 had an allocation amount that was not reported. The request letter for this particular disaster cited economic impacts of $53.8–94.2M in 2019 USD, and given government policy that the federal share cannot exceed 75% of a disaster request, this request equates to a maximum federal allocation of approximately $40.4–70.7M. Therefore, to estimate the total dollar amount allocated by Congress from 1994-2020, we combined the federal allocation amounts from the 65 known cases reported on the portal, the overall amount that was Congressionally approved for all disasters in aggregate in 2019 and 2020, and the estimate from the single unknown case (disaster number 19, Table S1).

To estimate the direct revenue impact associated with each fishery disaster—defined as revenue loss associated only with changes in landings and not throughout the supply chain—we evaluated each determination in accordance with NOAA Fisheries policy guidance on economic thresholds required for a successful determination (Denit, 2018); the difference in revenue of the impacted fishery during the disaster year(s) relative to the previous five-year average was calculated to produce revenue loss estimates. While revenue loss data were not explicitly reported in the federal disaster records, we were able to collect landings and nominal revenue data for each disaster using the NOAA Fisheries One-Stop Shop (FOSS) online database in most cases (NOAA, 2020d). When an approved disaster required finer spatial resolution landings and revenue data (e.g., if the fishery/area affected was smaller than the state-level scale), state landings sources were used to isolate regional-level data when possible. There were several disasters where landings and revenue data for the appropriate spatial scale (e.g., a regional bay) and/or management entity (e.g., tribal) were not obtainable; in these instances, the next best viable data source was used when available and the revenue impact estimate of the disaster was assigned a confidence level (i.e., low, medium, high) based on how well the landings and revenue data reflected the spatial scale of - and management entity impacted by - the disaster (Table S1). There were eight disasters in which viable data were completely unobtainable; these disasters are demarcated with “N/A” in the Net Revenue Change column in Table S1.

Direct revenue data were obtainable for 63 of the 71 approved fishery disasters. Changes in revenue in multi-year disasters—as reported on NOAA’s online portal (NOAA, 2020e)—were evaluated on an annual basis relative to the corresponding previous five-year average; while direct revenue data were obtainable for 63 disasters, calculating revenue loss for each disaster year resulted in more than 63 disaster years due to the handful of multi-year disasters (*n* = 79). Non-parametric bootstrapping techniques were used to estimate the sampling distribution of the median direct revenue impact and associated measures of uncertainty because the obtained revenue impact estimates were non-normally distributed (a Shapiro–Wilk test showed a significant departure form normality, *W* = 0.71, *p* < 0.001) . For each sample statistic, 100,000 replications were performed using the “Boot” package in R programming language (Davison & Hinkley, 1997; Canty & Ripley, 2019; R Core Team, 2019).

## Results

There were 96 Federal Fishery Disaster assistance requests filed in the US since 1994 (as of Oct 2020), spanning the impact period from 1989–2019, during which time 71 disasters were federally approved (Table S1). Congressional allocations and direct revenue loss data were available for 65 and 63 of the 71 approved disasters, respectively. The available data allowed estimates of the missing Congressional allocation values (see Supplementary Methods) and subsequent analyses of economic impact. Federal disaster records also allowed analyses of disaster frequency and causes of disasters.

### Economic impact

For the purposes of this study, all economic estimates are reflected in 2019 USD, with eleven 2020 assistance requests still pending. The total amount allocated by Congress throughout the entire federal disaster assistance program from 1994–2020 was over $1.99B (Table 2). This does not include six approved disasters with unreported federal assistance amounts, and it also does not include amounts that are still being determined for five disasters that were approved in 2019, as well as eleven disaster requests from 2019 and 2020 that have not yet been approved. However, a total of $165M was Congressionally appropriated for the 2019 fiscal year. In 2020, US Congress used the Coronavirus Aid, Relief, and Economic Security Act (CARES Act: https://www.fisheries.noaa.gov/national/noaa-fisheries-coronavirus-covid-19-update) to approve the allocation of an additional $300M for fisheries as a result of the ongoing COVID-19 pandemic.

**Table 2:** Total U.S. Congressional fishery disaster assistance (2019 USD) by cause and by federalfisheries management region. One additional disaster had an allocation amount that was not reported, but the request letter cited economic impacts of $53.8-94.2M. Anthropogenic causes include pollution and overfishing; environmental causes include marine heatwaves, harmful algal blooms, hurricanes, extreme drought, etc.; and a combination includes both anthropogenic and environmental causes. Examples of fisheries being impacted by a combination of causes can be found in some Pacific northwest salmon fishery disasters, which were caused by low returns that resulted from marine heatwaves, drought, disease, habitat impacts, mismanagement, and overfishing.

Cause	Alaska	Greater Atlantic	Pacific Islands	Southeast	West Coast	To be determined	Total
Anthropogenic	$82,000,000	$132,996,669		$30,940,000	$7,600,000		$253,536,669
Environmental	$174,292,189	$41,572,622	$1,140,000	$505,938,343	$170,723,211		$893,666,365
Combination of Both	$75,588,349	$36,600,000		$37,098,200	$281,802,589		$431,089,138
To be determined						$414,103,069	$414,103,069
Total	$331,880,538	$211,169,291	$1,140,000	$573,976,543	$460,125,800	$414,103,069	$1,992,395,241

Total assistance allocations were highly variable across space and time (i.e., no apparent trend), and ranged from $0–$300M annually (mean of $73.8M ± $96.1M SD). The Southeast Region (http://www.fisheries.noaa.gov/regions) obtained the most assistance since the inception of the program ($574M, 36.4% of overall determined funding), followed by the West Coast ($460M, 29.2%), Alaska ($332M, 21.0%), Greater Atlantic ($211M, 13.4%), and Pacific Islands ($1M, 0.1%). Overall, the entire western US (Alaska, West Coast, and Pacific Islands combined) accounted for 50.3% of all approved US disaster allocations (excluding pending disasters), while the Southeast and Greater Atlantic Regions accounted for 49.7% of allocations (Table 2).

We generated direct revenue loss estimates—defined as revenue loss associated only with changes in landings—for 63 of the 71 approved fishery disasters, which amounted to over $3.2B in revenue loss from 1994–2019, with additional significant revenue losses anticipated in 2020 due to the ongoing COVID-19 pandemic. These estimates were non-normally distributed, motivating the use of non-parametric statistical techniques. The median percent change in revenue during a disaster relative to the mean annual revenue over the preceding five-year period was −38.3 (IQR range = −70.4 –−1.3) across the 63 disasters for which data were attainable (Table S1). We also found that 12 of the 63 disasters showed a net *increase* in revenue in the year(s) of the disaster reported on the online portal. Omitting these 12 cases (to reflect total revenue losses that occurred) results in a median percent change in revenue of −60.6 (IQR range = −74.1 –−24.8). The median revenue loss in a disaster year was −$3.5M (*n* = 79), with a bootstrapped 95% confidence interval of [−$5.6 M –$4.8 M] (*n* = 100, 000 bootstrap samples) across the 63 disasters for which data were attainable. Omitting the 12 cases where revenue increased results in a median revenue loss in a disaster year of −$11.6M (*n* = 62), with a bootstrapped 95% confidence interval of [−$18.7 M –−$7.6 M] (*n* = 100,000 bootstrap samples). The Alaska Region accounted for 66.2% of the revenue impact overall, followed by the West Coast Region (18.1%), Southeast Region (10.7%), Greater Atlantic (5.1%), and Pacific Islands (<0.01%) (see GitHub repository for code specific to revenue loss estimates: https://github.com/vrsaccomanno/federal-fish-disasters.git).

### Disaster trends

**Figure 3:**
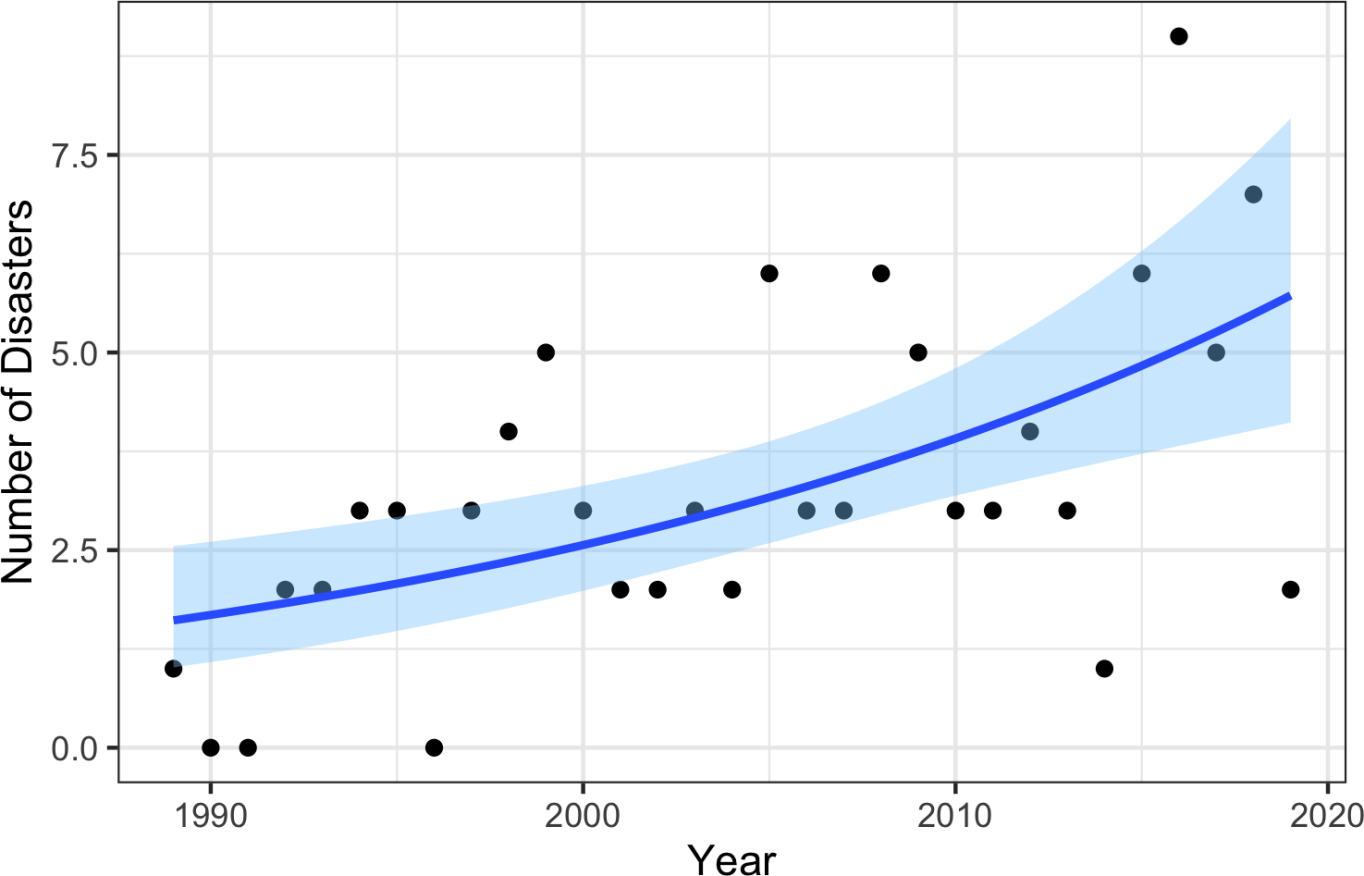
Model estimated change in the number of fishery disasters by impact year. The dark blue line represents the maximum likelihood fit (fixed effect of year; 4th row, Table 1), while the light blue shaded regions represent the 95% confidence intervals.

All statistical models (GitHub repository: https://github.com/vrsaccomanno/federal-fish-disasters.git) strongly indicate that disasters are increasing over time (i.e., that the number of disaster declarations and number of disasters per year have both increased; Table 1), even with eleven disasters during the 2017–2020 period that are still pending review (Fig. 3). The mixed effects models converged but with much larger AIC scores than counterpart fixed effects only models, indicating a poor model fit (Table 1). Moreover, the similarity in the estimated fixed year effect coefficients in both the mixed and fixed effects models (Table 1) suggests the mixed effects formulation is inconsequential to our findings.

It is possible that auto-correlation could manifest in the generative processes underlying our time-ordered data. That is, the underlying causes of disasters such as climate driven storm events or mis-management may carry momentum through time. Such a lack of independence between data points would violate the assumptions of the statistical methods we applied. However, we found no evidence of this based on an analysis of autocovariance in the residuals of the linear model regressing the number of disasters across years. In fact, the correlation at lag 1 (adjacent residuals) was ∼0, and no lag regardless of size had a correlation of >—0.-2— (https://github.com/vrsaccomanno/federal-fish-disasters.git). We are therefore confident that the statistical findings from our GLM models are robust.

Fishery disasters impacted every federal fisheries management region, and every coastal state in the U.S., as well as the US Virgin Islands, Puerto Rico, and American Samoa. Impacts were largely to commercial and recreational groundfish, commercial nearshore invertebrates, and commercial and Native American salmon fisheries, although several assistance requests and determinations simply stated only that “multiple fisheries” were impacted. Regional trends in disaster frequency showed a distinct shift from disasters across all regions between 1994–2015 to disasters occurring almost entirely in the West Coast and Southeast management regions from 2017–2019. The West Coast management region had the highest share of approved disasters (28/71), followed by the Southeast (18/71), Alaska (14/71), Greater Atlantic (10/41), and Pacific Islands (1/71) regions. Overall, the entire western US (including one approved disaster in the Pacific Islands) accounted for 60.6% of all approved US disasters, while the Gulf Coast and east coast accounted for 39.4% of approved disasters.

### Disaster causes

**Figure 4:**
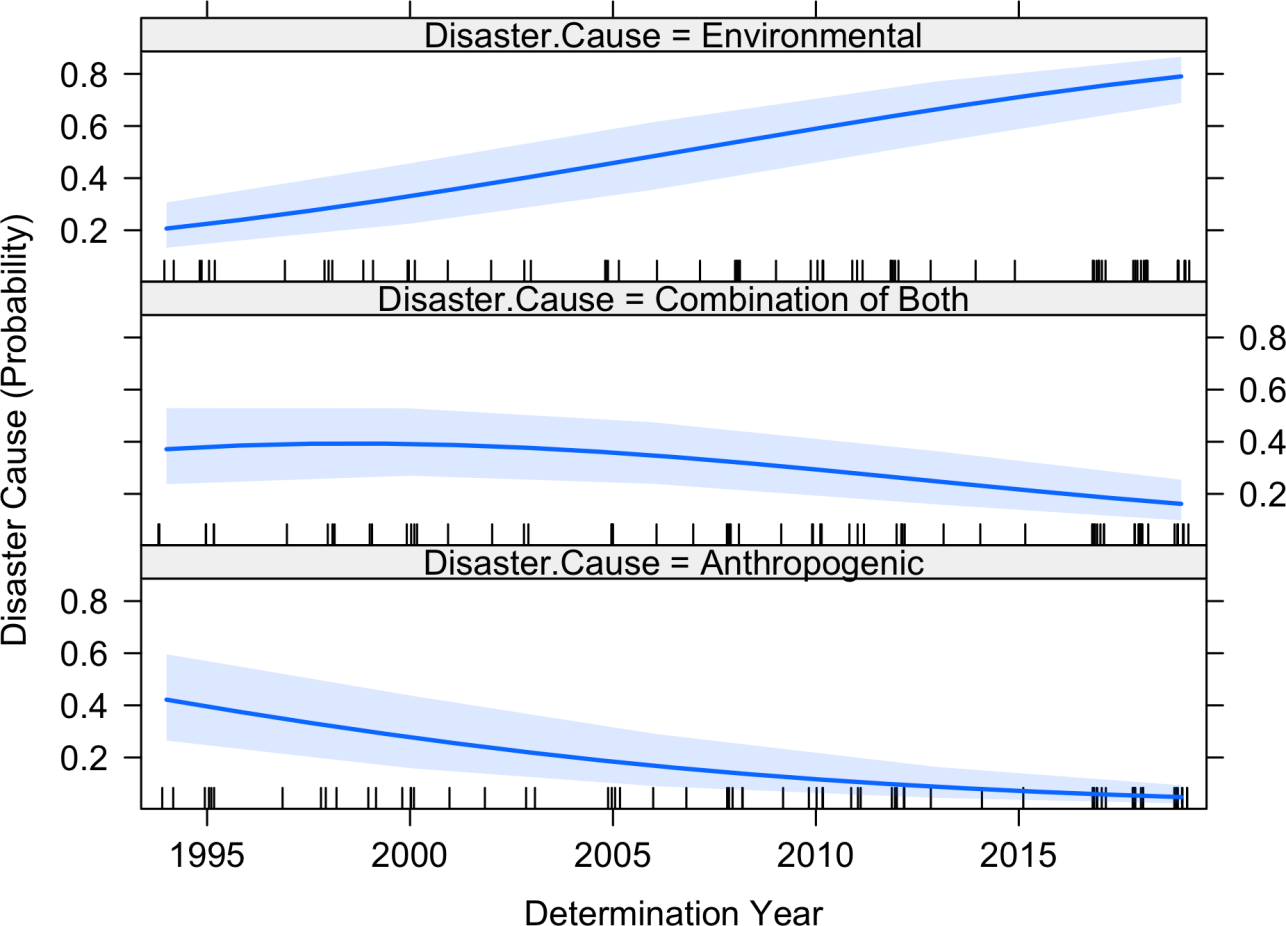
Model estimated change in the probability that a given disaster has roots in anthropogenic causes, environmental/anthropogenic combined causes, or environmental causes. The dark blue center lines represent the maximum likelihood fit, while the light blue shaded regions represent the 95% confidence intervals. The rug plot (tick marks, jittered around each year) at the bottom of each panel represents the number of declared disasters (of any cause) per year.

Since the 1990s, the predominant cause of fisheries disasters has shifted from anthropogenic to environmental in nature, with extreme environmental events reflecting 95.3% of the revenue loss during the most recent years (2014–2019), increasing from 38.5% during the first five years (1994–1998). Disaster causes were aggregated into three condensed categories: anthropogenic, environmental, or a combination of both. Marine heatwaves accounted for the most disasters overall, followed by hurricanes, overfishing, low returns (multiple Pacific salmon species), and HABs. Disasters caused by “low returns” in salmon fisheries were typically due to a combination of anthropogenic and environmental factors. Both the fixed and random effects logistic regression models performed similarly in terms of AIC scores (Table 1), with both showing significant time trends in the cause of disasters (Fig. 4). Regionally, marine heatwaves and HABs were often attributed to disasters in the both the West Coast and the New England / Greater Atlantic Regions, while hurricanes represented the primary cause of disasters in the Southeast Region. The majority of disasters with Congressional allocations were attributed to environmental causes ($894M, 56.6% of total determined allocations), followed by a combination of both ($431M, 27.3%), and lastly, anthropogenic causes ($254M, 16.1%; Table 2). There were no apparent trends in Congressional allocations by cause or region, although extreme environmental events were either partially or fully attributed to 84% of all Congressional allocations. In terms of revenue loss to fisheries, anthropogenic causes led to $1.8B (56.4%) in losses, although this was almost entirely driven by overfishing in the Alaska snow crab fishery (four federally approved disasters in total). Environmental causes resulted in an additional $900M (27.9%) in losses, and $505M (15.7%) in losses were attributed to a combination of anthropogenic and environmental causes (Table 3).

**Table 3:** Total revenue losses (2019 USD) by cause and by federal fisheries management region during disaster years.

Cause	Alaska	Greater Atlantic	Pacific Island	Southeast	West Coast	Total
Anthropogenic	$1,629,023,913	$156,252,205		$19,344,227	$16,669,638	$1,821,289,983
Environmental	$460,804,419	$7,525,113	$244,000	$319,003,223	$112,420,965	$899,997,721
Combination of Both	$46,055,192			$5,326,946	$453,935,310	$505,317,448
Total	$2,135,883,525	$163,777,318	$244,000	$343,674,396	$583,025,913	$3,226,605,152

### Federal timelines

The average time to fully process a disaster declaration was approximately 2.1 years (± 1.4 years SD), with a maximum total process time of six years, and one disaster request from 2005 that remains undetermined. The time period from the disaster occurrence to the formal assistance request filing averaged 6.4 months (± 12.0 months SD), with 66.2% of requests being filed within 1 year after the disaster, and 87.7% filed within 2 years after the disaster. Once the request was filed, determination time (time between steps 2 and 3 of the 6-step process) lasted as long as 4.0 years (mean of 8.3 ± 8.7 months SD). One disaster approval occurred 92 days before the request was formally filed (disaster number 29: Hurricane Katrina).

## Discussion

Federal Fisheries Disasters have generated large, yet remarkably variable revenue impacts throughout the US during the last three decades, and the frequency of disasters is increasing over time. In turn, the US government has spent billions of dollars to mitigate these impacts. In the early days of fishery disaster determinations, these impacts were largely due to mixed factors and relatively poor fisheries management. Fortunately, such direct anthropogenic causes of disasters appear to be on the wane as fisheries management improves (e.g., Marshall et al., 2019; Hilborn et al., 2020; NOAA, 2020b). However, environmentally-driven fishery disasters are clearly on the rise, fueled by extreme environmental events that occur as symptoms of a changing climate (Cheung & Frölicher, 2020; Jacox et al., 2020; Laufkötter, Frölicher & Zscheischler, 2020). For example, the US west coast marine heat wave event of 2013–2016 (e.g., the warm ‘blob’) produced a broad range of coastwide impacts (Cavole et al., 2016), including several approved Federal Fishery Disasters (e.g., Holland & Leonard, 2020). In total, these marine heatwaves were the dominant environmental cause of Federal Fishery Disasters nationwide in the 30-year time series, followed by hurricanes and HABs, but almost all marine heatwave events occurred only in the recent 2013–2020 period. This recent emergence of marine heatwaves, which occurred on both the east and west coasts of the U.S., thus accounted for much of the increasing trend in fishery disasters. Taken together, our findings suggest that not only are fisheries disasters on the rise, but the linkage between climate change, marine heatwaves (Frölicher, Fischer & Gruber, 2018; Oliver et al., 2018; Holbrook et al., 2019), and now Federal Fishery Disasters is becoming increasingly clear, and the causes of fishery disasters are shifting from those with conventional management solutions, to those that require more pro-active and climate-ready policies (Pinsky & Mantua, 2014). Similar temporal shifts in impact causes were documented for animal mass mortality events, which included both marine and terrestrial species (Fey et al., 2015). This shift toward increased impacts due extreme environmental events is also consistent with numerous studies documenting unprecedented and accelerating oceanographic, ecosystem, and fisheries changes during the same time period (Bond et al., 2015; Peterson, Robert & Bond, 2015; Ritzman et al., 2018; Thompson, 2018; Arafeh-Dalmau et al., 2019; Piatt et al., 2020; Santora et al., 2020).

Despite the multi-billion dollar economic impacts, we are likely underestimating the true magnitude of the economic effects of Federal Fishery Disasters. First, our economic analyses only include estimates for 63 of the 71 approved fishery disasters due to data limitations, and there are still eleven pending disaster requests that were also not included in the analysis. Second, our analyses represent fisheries that were formally included in federal assistance requests, but there may be impacted fisheries/communities that never applied for assistance. Third, our revenue impact estimates only included losses during the disaster year(s) as reported on the online portal, not potential losses in subsequent years due to delayed impacts. Fourth, our impact analyses were based on the NOAA disaster determination model that uses direct commercial fishing revenue as the primary impact metric, but indirect economic losses across supply chains are undoubtedly larger (Holland & Leonard, 2020). In addition, direct and indirect revenue contributions from recreational fisheries, which generate $68B in national annual sales (NOAA, 2018), were not included in our economic impact analyses due to data limitations. Fifth, because disaster requests and approvals often take years to process and publicly report, there will likely be disaster requests added to the most recent 2018-2020 period, meaning that our results may represent a conservative estimate of increasing trends over time and economic impacts during these recent years. Finally, our study only focused on economic impact estimates, but fishery disasters also reflect impacted ecosystems, communities, and cultures that were not the focus of our analyses (e.g., Smith & Gilden, 2000). These represent critical components of the overall impact from fishery disasters that should be a focus of future science and policy engagements.

The magnitude of economic impacts of fishery disasters is clearly a problem, but the lack of standardized reporting with sufficient detail in disaster requests and determinations creates significant challenges for implementing solutions. For example, the median revenue loss across all approved disasters (38.3% loss) was notably less than the 80% threshold that triggers federal determination of a commercial fishery failure, and is just above the 35% threshold that allows further review according to federal guidelines. We also identified 12 cases of federally approved disasters in which fishery-specific direct revenue actually increased during the disaster year relative to the previous 5-year average, a surprising finding given that an increase in revenue in a disaster year does not meet NOAA Fisheries policy guidance on economic thresholds required for a successful determination (we omitted these cases from our impact estimates under the rationale that gains in one fishery do not erase losses sustained in another). Our conservative estimates of economic impacts may not be capturing the full scale of impact, but the consistency in the economic metric used between our study and the federal determination process suggests a need for improved economic data collection and federal reporting. Improved reporting would also provide critically needed clarification about specifically which fisheries are impacted. For example, fishery disasters ranged in spatial scale from individual rivers to multiple states; they might impact only one Native American community or several; and they might affect only one fishery or virtually every fishery in a state or region. Enumerating disaster impact by specific fisheries is thus problematic because information about which species, fisheries, and communities were affected was not always provided in federal disaster assistance requests and determinations. These details could facilitate improved estimations of impact, more transparency when and where the Secretary of Commerce determines there are special circumstances that may justify using a lower threshold of percent revenue loss in disaster determinations, and a more streamlined and transparent process for estimating and allocating assistance to the most heavily impacted fisheries and fishing communities.

Problems also exist with assistance processing lags and opaque determination process. Across all Federal Fishery Disaster declarations, total federal process duration (Fig. 1, duration between steps one and six) averaged 2.1 years, ranging from zero to six years after the disaster event. Such delayed response times are likely due both to the complex determination and allocation process, and the burden of increasing disaster assistance request rates. The administrative process can also be contentious because federal assistance timelines can significantly lag impact events, federal decision-making is often unclear (or undocumented), impacted individuals or entities may be difficult to identify, and the degree of economic impacts may be difficult to quantify (Upton, 2019). A more detailed accounting of determinations could address these issues, and reveal aspects of the determination process that are leading to such highly variable response times.

The federal response to the growing impacts of fishery disasters clearly needs reform, but the lack of standardized reporting in Federal Fishery Disaster requests and determinations makes identifying solutions difficult. Our finding that many of the published determinations failed to list the specific commercial fishery(ies) used to make the determination is problematic. While disasters ranged in scale from individual fish stocks and fisheries to multiple states and fishing communities, the details provided in determination documents do not reflect the complexity of disaster impacts. Transparent, detailed, and mechanistic descriptions of economic impact determinations are necessary for the Secretary of Commerce to determine if there are special circumstances that may justify using a lower threshold of percent revenue loss in Federal Fishery Disaster determinations. To achieve this, public reporting of a full economic impact assessment in each fishery would undoubtedly cause prohibitive slowing of the disaster assistance process, but publicly reporting the data that are already behind each disaster determination would achieve a greater level of transparency and impact detail while adding little to the process and timeline. In addition, there is need for a more streamlined, fair, and transparent process for allocating assistance (Oliver, 2019; Upton, 2019). While compensation of fishing communities is the ultimate goal of disaster assistance, we found that approximately 40% of federal determinations failed to identify the specific fishery(ies) impacted by the disaster, making any future efforts to accountability in assistance allocation difficult, if not impossible.

This study represents a national-scale view of fishery disasters, a problem that has worsened over time, and has cascading socioeconomic impacts to society. This study also highlights a clear need for consideration of ecosystem and cultural impacts of fishery disasters that were not the focus of this study. For example, Native American fisheries represent a far more extensive history of coastal harvest and culture than commercial or recreational fisheries, illustrating the intimate connection possible between nature and people that span millennia. However, in three decades, 29 federally approved (and four pending) disasters have impacted salmon fisheries in the Pacific northwest (Smith & Gilden, 2000). Over half of these requests came from Native American communities that have been intimately connected with salmon populations for generations, yet limited syntheses of disaster impacts to indigenous communities are available. Knowledge of the disruptions in such cultural relationships should be a necessary part of efforts to mitigate the totality of fishery disaster impacts, yet such considerations are not currently part of the Federal Fishery Disaster determination process.

As currently structured, the legal framework for declaring Federal Fishery Disasters can depend on a range of economic impact metrics that typically go unreported in disaster determinations, but Congressional appropriations depend on demonstrating a direct loss of revenue to commercial fisheries. However, there is no question that the broader market and non-market value of fisheries losses grossly outweigh the direct revenue loss (Holland & Leonard, 2020; Seung, 2017). These additional impacts encapsulate fisheries supply chains, cultural values, and non-commercial impacts. The fact that the existing legal mechanisms for fishery disaster assistance hinge largely on direct revenue loss may explain why many declared Federal Fishery Disasters fail to meet the defined economic loss thresholds (e.g., in some disasters, revenue actually increased); it is likely lawmakers are making a good-faith effort to account for the true breadth of economic and societal loss stemming from disaster. However, the lack of a codified mechanism for doing so contributes to inaccurate accounting of financial assistance.

## Conclusions

As disaster impacts to fishing communities worsen via increased disaster frequencies, federal response times to mitigate these impacts continue to lag by years in most cases (Upton, 2019). Developing proactive, rather than reactive, mitigation strategies may partially address this problem. Recent shifts in the causes of fishery disasters toward extreme environmental events suggest a need to explore incentive structures for linking disaster assistance with proactive climate-ready fisheries management (Pinsky & Mantua, 2014). Regional fishery disaster vulnerability assessments (Badjeck et al., 2013), ecosystem-based fishery management approaches (Holsman et al., 2020), responsive harvest control rules (Kritzer et al., 2019), and accounting for climate-related shifts in fisheries productivity/distributions (Free et al., 2020) may all be useful in guiding science and management efforts toward this end. Industry insurance mechanisms, such as those trialed in west coast salmon fisheries, might also mitigate future extreme event scenarios by providing timely assistance to those in greatest need, as well as incentivized support for climate-ready fisheries management goals (Mumford et al., 2009).

There has never been a better time to identify and implement more effective government response to disasters. The COVID-19 pandemic has added a new layer of impacts across virtually every sector of the global economy, including fisheries (Bennett et al., 2020). By September 2020, the US had already reached a record annual number of natural disasters declared (56% of which were linked to COVID-19 at the time of submission) by FEMA in the history of US natural disaster declarations (1953-2020). In the fisheries sector, the seafood industry landscape has been severely impacted, with entire fleets tied to the docks, and fisheries market closures occurring around the world (Bennett et al., 2020). The pandemic has led to an unprecedented need for federal assistance across a significant portion of the US economy, including $300M approved for fishery disasters in 2020 as part of the Coronavirus Aid, Relief, and Economic Security Act (CARES Act). The scale of need for federal assistance this year highlights the critical importance of preventing and mitigating disasters with climate-ready management models, maximizing efficiency in the federal assistance process, and increasing the impact of every federal dollar spent during these extreme and uncertain circumstances.

##  Supplemental Information

10.7717/peerj.11186/supp-1 Supplemental Information 1 Summary of Federal Fishery Disaster economic impact findings for approved disasters

## Additional Information and Declarations

### Data Availability

The following information was supplied regarding data availability: All federally-declared fishery disaster data used in these analyses are available at NOAA: https://www.fisheries.noaa.gov/national/funding-and-financial-services/fishery-disaster-determinations
All code and fishery landings and revenue data are available in Github: https://github.com/vrsaccomanno/federal-fish-disasters.git
Data was taken from the NOAA Fisheries One Stop Shop (FOSS) website: https://foss.nmfs.noaa.gov
Fishery disaster trend analyses were performed using the data available as a Supplemental File.
