# Supplementary material for: The rise in climate change-induced federal fishery disasters in the United States
Source: PeerJ. 2021 Apr 22;9:e11186. doi: 10.7717/peerj.11186 (PMC8071068; doi:10.7717/peerj.11186)
Supplement: Supplemental Information 1 [file peerj-09-11186-s001.docx]

Table S1. Summary of Federal Fishery Disaster economic impact findings for approved disasters; “Confidence in Revenue Data” describes how well the landings and revenue data obtained for each disaster fits both the spatial scale of the disaster, as well as the management entity impacted by the disaster. Disasters 88 and 91-96 are not shown because they are still pending (as of Aug 21, 2020).

| **Disaster Number** | **Fishery/ (ies)** | **Management Zone** | **Determination Year** | **Disaster Cause** | **Appropriation (2019 USD)** | **Net Revenue Change (2019 USD)** | **Confidence in Revenue Data** | **Revenue Data Source(s)/ Notes*** |
| --- | --- | --- | --- | --- | --- | --- | --- | --- |
| 90 | Pacific Sardine | West Coast | 2019 | Envr | $2,263,282 | ($1,687,134) | Medium | NOAA FOSS; no 2018 or 2019 data |
| 89 | Multi spp | Southeast | 2019 | Envr |  | N/A |  |  |
| 87 | Red Sea Urchin | West Coast | 2019 | Envr | $3,395,930 | ($4,020,219) | High | CDFW, PacFIN |
| 86 | Chinook Salmon | West Coast | 2019 | Combo | $2,270,589 | N/A |  |  |
| 85 | Sockeye Salmon | Alaska | 2019 | Combo | $10,533,580 | ($12,785,858) | Medium | Request letter; data is thorough |
| 84 | Multi spp | Southeast | 2018 | Envr | $8,037,120 | ($17,602,000) | Medium | 2018 data not available through NOAA FOSS; data is from NCDMF |
| 83 | Multi spp | Southeast | 2018 | Envr | $8,124,480 | ($13,478,043) | High | FFWCC |
| 82 | Penaeid Shrimp (Multi spp) | Southeast | 2019 | Envr | $7,528,781 | ($392,817) | High | GDNR and NCDENR |
| 81 | Pacific Cod | Alaska | 2019 | Combo | $24,904,769 | ($1,265,553) | High | ADFG for only the gulf |
| 80 | Coho and Chinook | West Coast | 2018 | Envr | $1,720,160 | ($1,702,615) | Low | Request letter |
| 79 | Multi spp | Southeast | 2018 | Envr | $14,503,074 | $8,567,582 | High | NOAA FOSS; revenue increase |
| 78 | Coho Salmon | West Coast | 2018 | Envr | $1,008,800 | ($450,486) | Low | Determination letter; unclear if "economic loss" is only langings |
| 77 | Multi spp | Southeast | 2018 | Envr | $69,407,687 | ($112,360,000) | High | NOAA press release |
| 75 | Pacific Sardine | West Coast | 2018 | Envr | $1,705,600 | ($6,857,471) | High | NOAA FOSS |
| 74 | Chinook Salmon | West Coast | 2018 | Envr | $9,241,440 | ($24,689,000) | Low | OR and CA request letter; no tribal data |
| 73 | Coho and Pink Salmon | West Coast | 2018 | Envr | $4,010,240 | ($1,736,733) | Low | Data taken from 3 out of 7 request letters and extrapolated out |
| 72 | Pink Salmon | Alaska | 2017 | Envr | $59,743,012 | ($121,986,123) | Medium | NOAA FOSS; includes the Yukon management area |
| 71 | Ocean Salmon Troll | West Coast | 2017 | Envr | $884,465 | ($1,092,398) | High | PFMC 2018 Review of Ocean Salmon Fisheries |
| 70 | Chinook Salmon | West Coast | 2017 | Envr | $4,096,798 | N/A |  |  |
| 69 | Dungeness Crab | West Coast | 2017 | Envr | $1,570,071 | ($3,300,000) | Low | Request letter; did not provide 5-year revenue, but did specify direct revenue loss |
| 68 | Coho, Chinook, Chum Salmon | West Coast | 2017 | Envr | $14,355,181 | ($743,741) | Low | Request letter; no economic estimate from Squaxin or Nisqually; estimate from Port Gamble or S'Klallam |
| 67 | Dungeness and Rock Crab | West Coast | 2017 | Envr | $27,345,104 | ($38,543,341) | High | CDFW and value from PacFIN |
| 66 | Salmon (Multi spp) | West Coast | 2017 | Envr | $15,483,355 | ($5,521,606) | Low | NOAA FOSS; data does not isolate Willapa and Grays; no tribal estimate |
| 65 | Sockeye Salmon | West Coast | 2017 | Envr | $2,997,584 | N/A |  |  |
| 64 | White Shrimp | Southeast | 2015 | Combo | $1,168,200 | ($4,402,333) | High | NOAA FOSS |
| 63 | Sockeye Salmon | West Coast | 2014 | Envr | $2,125,200 | ($4,463,200) | High | NOAA press release |
| 61 | Multi spp | Greater Atlantic | 2012 | Envr | $8,944,622 | $18,876,191 | High | NOAA FOSS; revenue increase |
| 59 | Oyster | Southeast | 2013 | Envr | $7,074,517 | $3,380,401 | High | FFWCC; Commercial Fisheries Landings Summaries report creator. Revenue increase. |
| 58 | Chinook Salmon | Alaska | 2012 | Envr | $23,709,177 | ($3,552,073) | Medium | ADFG; isolated by region but not tribal |
| 56 | Groundfish (Multi spp) | Greater Atlantic | 2012 | Anthro | $37,446,669 | ($12,464,203) | Medium | NOAA FOSS; in-house species selection |
| 55 | Multi spp | Southeast | 2012 | Envr | $12,473,684 | $9,499,690 | High | NOAA FOSS; revenue increase |
| 53 | Salmon (Multi spp) | West Coast | 2011 | Combo |  | ($548,565) | Medium | ODFW for all ports south of Cape Falcon |
| 51 | Multi spp | Pacific Island | 2012 | Envr | $1,140,000 | ($244,000) | High | Federal Register |
| 50 | Salmon (Multi spp) | West Coast | 2010 | Combo |  | N/A |  |  |
| 49 | Multi spp | Southeast | 2010 | Anthro | $30,940,000 | ($19,344,227) | High | NOAA FOSS |
| 47 | Sockeye Salmon | West Coast | 2011 | Combo | $2,340,000 | N/A |  |  |
| 46 | Shellfish (Multi spp) | Greater Atlantic | 2010 | Envr |  | ($5,798,530) | Medium | NOAA FOSS; in-house species selection |
| 45 | Chinook Salmon | Alaska | 2010 | Combo | $5,950,000 | ($1,536,772) | High | ADFG; isolated by region |
| 43 | Salmon (Multi spp) | West Coast | 2009 | Combo |  | ($15,479,882) | High | NOAA FOSS |
| 40 | Shellfish (Multi spp) | Greater Atlantic | 2008 | Envr | $6,100,000 | $6,239,125 | Medium | NOAA FOSS; in-house species selection |
| 39 | Multi spp | Southeast | 2008 | Envr | $57,340,000 | $21,304,713 | High | NOAA FOSS |
| 38 | Sockeye Salmon | West Coast | 2008 | Combo | $2,440,000 | N/A |  |  |
| 37 | Blue Crab | Greater Atlantic | 2008 | Combo | $36,600,000 | $14,827,134 | Medium | NOAA FOSS; does not isolate Chesapeake Bay |
| 36 | Salmon (Multi spp) | West Coast | 2008 | Combo | $207,400,000 | ($11,888,830) | Medium | NOAA FOSS; "rainbow trout" for steelhead. Did not isolate tribal |
| 33 | Snow Crab | Alaska | 2011 | Anthro | $11,700,000 | $73,954,209 | Medium | ADFG; does not isolate just Bering Sea |
| 31 | Snow Crab | Alaska | 2007 | Anthro | $12,700,000 | ($1,126,347) | Medium | ADFG; does not isolate just Bering Sea |
| 30 | Salmon (Multi spp) | West Coast | 2006 | Envr | $78,520,000 | ($541,845) | High | SWFSC technical report on the Klamath |
| 29 | Multi spp | Southeast | 2005 | Envr | $287,550,000 | ($169,681,222) | High | NOAA FOSS |
| 27 | Shellfish (Multi spp) | Greater Atlantic | 2005 | Envr | $2,700,000 | ($1,378,229) | Medium | NOAA FOSS; in-house species selection |
| 26 | Shellfish (Multi spp) | Greater Atlantic | 2005 | Envr | $2,700,000 | $20,118,331 | Medium | NOAA FOSS; in-house species selection |
| 25 | Snow Crab | Alaska | 2005 | Anthro | $13,500,000 | ($787,346,805) | Medium | ADFG; does not isolate just Bering Sea |
| 23 | Blue Crab | Southeast | 2003 | Combo | $7,100,000 | ($924,613) | High | NOAA FOSS |
| 22 | Snow Crab | Alaska | 2003 | Anthro | $14,200,000 | ($239,347,664) | Medium | ADFG FOSS; does not isolate just Bering Sea |
| 21 | Snow Crab | Alaska | 2001 | Anthro | $14,700,000 | ($289,342,969) | Medium | ADFG FOSS; does not isolate just Bering Sea |
| 19 | Sockeye Salmon | West Coast | 2002 | Combo |  | ($14,130,000) | Low | Request letter - specifies loss as "direct benefits"; assumed total over multi-year period |
| 18 | Salmon (Multi spp) | Alaska | 2000 | Combo | $34,200,000 | ($30,467,010) |  |  |
| 17 | Snow Crab | Alaska | 2000 | Anthro | $15,200,000 | ($300,618,829) | High | ADFG |
| 15 | Multi spp | Southeast | 1999 | Envr | $9,263,000 | $10,067,473 | Medium | NOAA FOSS; in-house species selection |
| 14 | American Lobster | Greater Atlantic | 2000 | Envr | $21,128,000 | ($348,354) | High | NOAA FOSS |
| 13 | Groundfish (Multi spp) | West Coast | 2000 | Anthro | $7,600,000 | ($16,669,638) | Medium | PacFIN Commercial Groundfish Catch Report for All Gear Types and All Areas |
| 12 | Spiny Lobster and Stone Crab | Southeast | 1999 | Envr | $7,536,000 | ($5,489,142) | High | FFWCC |
| 11 | Salmon (Multi spp) | Alaska | 1998 | Envr | $79,500,000 | ($143,208,616) | High | ADFG |
| 10 | Salmon (Multi spp) | West Coast | 1998 | Combo | $17,490,000 | ($19,862,009) | High | NOAA FOSS |
| 9 | Brown Shrimp | Southeast | 1998 | Combo | $3,180,000 | $45,284,096 | High | NOAA FOSS |
| 7 | Salmon (Multi spp) | Alaska | 1997 | Envr | $11,340,000 | ($192,057,608) | High | ADFG |
| 6 | Salmon (Multi spp) | West Coast | 1995 | Combo | $22,230,000 | ($30,668,064) | High | NOAA FOSS |
| 5 | Groundfish (Multi spp) | Greater Atlantic | 1995 | Anthro | $42,750,000 | ($74,261,441) | Medium | NOAA FOSS; no American Plaice |
| 4 | Multi spp | Southeast | 1995 | Combo | $25,650,000 | $139,911,239 | High | NOAA FOSS |
| 3 | Salmon (Multi spp) | West Coast | 1994 | Combo | $27,632,000 | ($361,357,960) | High | NOAA FOSS |
| 2 | Groundfish (Multi spp) | Greater Atlantic | 1994 | Anthro | $52,800,000 | ($58,047,168) | Medium | NOAA FOSS; no American Plaice |
| 1 | Multi spp | Southeast | 1995 | Envr | $17,100,000 | N/A |  |  |
